# Human Norovirus Replication in Human Intestinal Enteroids as Model to Evaluate Virus Inactivation

**DOI:** 10.3201/eid2408.180126

**Published:** 2018-08

**Authors:** Veronica Costantini, Esther K. Morantz, Hannah Browne, Khalil Ettayebi, Xi-Lei Zeng, Robert L. Atmar, Mary K. Estes, Jan Vinjé

**Affiliations:** Centers for Disease Control and Prevention, Atlanta, Georgia, USA (V. Costantini, J. Vinjé);; Synergy America, Inc., Atlanta (E.K. Morantz);; Oak Ridge Institute for Science and Education, Oak Ridge, Tennessee, USA (H. Browne);; Baylor College of Medicine, Houston, Texas, USA (K. Ettayebi, X.-L. Zeng, R.L. Atmar, M.K. Estes)

**Keywords:** norovirus, human intestinal enteroids, inactivation, viruses, replication, virus inactivation

## Abstract

Human noroviruses are a leading cause of epidemic and endemic acute gastroenteritis worldwide and a leading cause of foodborne illness in the United States. Recently, human intestinal enteroids (HIEs) derived from human small intestinal tissue have been shown to support human norovirus replication. We implemented the HIE system in our laboratory and tested the effect of chlorine and alcohols on human norovirus infectivity. Successful replication was observed for 6 norovirus GII genotypes and was dependent on viral load and genotype of the inoculum. GII.4 viruses had higher replication levels than other genotypes. Regardless of concentration or exposure time, alcohols slightly reduced, but did not completely inactivate, human norovirus. In contrast, complete inactivation of the 3 GII.4 viruses occurred at concentrations as low as 50 ppm of chlorine. Taken together, our data confirm the successful replication of human noroviruses in HIEs and their utility as tools to study norovirus inactivation strategies.

Human noroviruses are the leading cause of epidemic and endemic acute gastroenteritis worldwide (*1*). A major barrier to studying the pathogenesis, virus–host interactions, and effect of control measures to prevent and treat norovirus gastroenteritis has been the lack of a robust and reproducible cell culture system. Since the discovery of norovirus in 1972, many research groups have attempted to grow human norovirus. Initial studies included a comprehensive number of primary and continuous cell lines that had been used successfully to grow other viruses, but none supported human norovirus replication (*2*). Other attempts included the use of a differentiated human embryonic small-intestinal cell line (INT 407) (*3*), but the results could not be confirmed by others (*4**–**6*). The discovery of murine norovirus (MNV) in 2003 and the fact that this virus successfully replicated in a murine macrophage cell line in vitro and in primary immune cells in vivo suggested that immune cells may also support replication of human norovirus (*7*). However, immune cells isolated from healthy adults did not support replication (*8*). A more recent study reported that BJAB cells, a continuous human B cell line, supported replication of a GII.4 norovirus strain in the presence of bacteria (*9*). Although initially promising, these results have not been consistently confirmed by other groups (*10*).

The first steps toward a new cell culture system for human norovirus included the detection of human norovirus antigen in duodenal and jejunal enterocytes in tissue sections from human norovirus–infected transplant patients (*11*) and the development of human intestinal enteroids (HIEs) derived from nontransformed small intestine and colonic tissues (*12*). The HIEs, or “mini-guts,” are generated from stem cells present in the intestinal crypts isolated from human intestinal tissue and cultured indefinitely as ex vivo, 3-dimensional (3D) cultures in growth-factor–enriched media (*13**–**15*). HIE cultures recapitulate the complexity and cell diversity of the gastrointestinal tract in relatively the same proportions as in the intestine itself (*14**,**15*) and successfully support the replication of human rotavirus (*13*) and human norovirus (*16**,**17*). These studies confirmed the role of enterocytes as the major site for human norovirus replication and host restriction based on genetic factors, as well as the role of bile as a strain-specific requirement or enhancer for virus infectivity.

During the past 40 years, the efficacy of inactivation and disinfection procedures for human norovirus could be evaluated only by human challenge studies (*18**,**19*) or by using cultivable surrogate viruses such as MNV, feline calicivirus, or Tulane virus (*20**–**23*). However, without direct confirmation that any of these surrogates correlate with inactivation of infectious human norovirus, no consensus has been reached on the best surrogate for human norovirus (*24**,**25*). In this study, we demonstrate the successful implementation of HIE cultures, show successful replication of several human norovirus genotypes, and demonstrate the applicability of HIEs to evaluate the efficacy of chlorine and alcohols on reducing virus infectivity.

## Materials and Methods

Detailed methods and description of HIE cultures, gene expression analysis, viral infections, norovirus detection, and statistical analyses are provided in the Technical Appendix. This investigation was determined by the Centers for Disease Control and Prevention (CDC) to be public health nonresearch and therefore not subject to institutional review board review.

### Fecal Samples

We included 80 human norovirus-positive fecal samples (12 genogroup [G] I, 65 GII, and 3 GIV) collected during 2000–2017 in the study (Table 1). Samples were stored at 4°C or −70°C from collection time until the time of testing. All samples were tested during April 2016–December 2017.

**Table 1:** Human norovirus–positive fecal samples tested on 80 jejunal HIEs in study of human norovirus replication in HIEs

Genotype*		No. samples	Mean norovirus RNA copies/μL	Patient age group		Collection date†	Storage condition‡	Outbreak or sporadic	No. inactivated/ no. samples %
Capsid	RdRp	Years	No.
GI.1	GI.P1	4	0.5–19.3 × 10^3^	>18	4	2000	−70°C	Sporadic	0/4 (0)
GI.3	GI.P3	2	0.3–59.4 × 10^4^	0–12	1	2013 Jul	−70°C	Sporadic	0/4 (0)
				>18	1	2005	−70°C	Outbreak	
	GI.Pd	2	1.2–8.7 × 10^4^	0–12	1	2015 Nov	−70°C	Sporadic	
					1	2017 Feb	−70°C	Sporadic	
GI.4	GI.P4	1	2.2 × 10^2^	>18	1	2000	−70°C	Outbreak	0/1 (0)
GI.7	GI.P7	3	2.6–16.4 × 10^3^	0–12	1	2010 Nov	−70°C	Sporadic	0/3 (0)
					1	2014 Mar	−70°C	Sporadic	
					1	2016 Dec	−70°C	Sporadic	
GII.1	GII.Pg	1	1.4 × 10^5^	0–12	1	2017 May	−70°C	Sporadic	1/1 (100)
GII.2	GII.P16	3	0.2–52.6 × 10^3^	0–12	2	2017 Feb	−70°C	Sporadic	1/2 (50)
					1	2017 Mar	−70°C	Sporadic	0/1 (0)
GII.3	GII.P21	2	1.0–6.4 × 10^5^	0–12	1	2012 Mar	−70°C	Sporadic	0/1 (0)
					1	2015 May	−70°C	Sporadic	
	GII.P12	2	1.6–4.4 × 10^6^	0–12	2	2012 Aug	−70°C	Sporadic	1/2 (50)
	GII.P16	4	0.2–141.1 × 10^4^	0–12	1	2012 Mar	−70°C	Sporadic	0/4 (0)
					1	2012 Jun	−70°C	Sporadic	
					1	2015 Oct	−70°C	Sporadic	
					1	2016 Dec	−70°C	Sporadic	
GII.4 Den Haag	GII.P4 Den Haag	3	1.3–161.6 × 10^4^	>18	1	2010 May	4°C	Outbreak	0/1 (0)
				0–12	1	2013 Jun	−70°C	Sporadic	0/1 (0)
					1	2015 May	−70°C	Sporadic	1/1 (100)
GII.4 New Orleans	GII.P4 New Orleans	1	4.1 × 10^5^	0–12	1	2013 Apr	−70°C	Sporadic	0/1 (0)
GII.4 Sydney	GII.Pe	22	3.5 × 10^3^–2.1 × 10^7^	0–12	3	2012	−70°C	Sporadic	1/3 (30)
					1	2015 Jan	−70°C	Sporadic	1/1 (100)
					7	2015 Feb	−70°C	Sporadic	3/7 (40)
					1	2015 Mar	−70°C	Sporadic	1/1 (100)
					1	2015 Apr	−70°C	Sporadic	1/1 (100)
					1	2015 Oct	−70°C	Sporadic	0/1 (0)
					1	2016 Apr	−70°C	Sporadic	0/1 (0)
				>18	1	2011	−70°C	Outbreak	0/1 (0)
					1	2013	−70°C	Outbreak	0/1 (0)
					1	2012 May	4°C	Outbreak	0/1 (0)
					2	2016 Apr	4°C	Outbreak	1/2 (50)
					2	2016 May	4°C	Outbreak	0/2 (0)
	GII.P16	13	0.4 × 10^3^–6.1 × 10^6^	0–12	2	2016 Dec	−70°C	Sporadic	1/2 (50)
					6	2017 Feb	−70°C	Sporadic	0/6 (0)
					5	2017 Mar	−70°C	Sporadic	0/5 (0)
	GII.P4 New Orleans	4	0.6–14.9 × 10^4^	0–12	1	2015 Jan	−70°C	Sporadic	0/4 (0)
					1	2017 Jan	−70°C	Sporadic	
					1	2017 Feb	−70°C	Sporadic	
					1	2017 Mar	−70°C	Sporadic	
GII.5	GII.P22	1	1.1 × 10^4^	0–12	1	2010 Nov	−70°C	Sporadic	0/1 (0)
GII.6B	GII.P7	1	7.6 × 10^4^	0–12	1	2015 Jan	−70°C	Sporadic	0/1 (0)
GII.6	GII.P7	4	0.1–8.4 × 10^6^	0–12	1	2012 Dec	−70°C	Sporadic	0/4 (0)
					2	2015 Jan	−70°C	Sporadic	
					1	2017 Jan	−70°C	Sporadic	
GII.7	GII.P7	2	0.3–9.1 × 10^6^	>18	1	2010 Oct	−70°C	Sporadic	0/2 (0)
				0–12	1	2012 Aug	−70°C	Sporadic	
GII.14	GII.P7	1	6.1 × 10^4^	0–12	1	2016 Dec	−70°C	Sporadic	1/1 (100)
GII.17	GII.Pe	1	3.4 × 10^5^	0–12	1	2010 Oct	−70°C	Sporadic	1/1 (100)
GIV	GIV.P1	3	0.3–13.6 × 10^3^	>18	3	2016 May	4°C	Outbreak	0/3 (0)
Total		80			80				16/80 (20)

*Dual genotyping based on sequencing partial RdRp and capsid regions (*26**,**27*). HIE, human intestinal enteroids; RdRp, RNA-dependent RNA polymerase. †When month was not available, only year of collection is reported. ‡Samples were stored at the indicated temperature.

#### Human Intestinal Enteroid Culture

Baylor College of Medicine (Houston, TX, USA) provided secretor-positive jejunal HIE cultures (J2 and J3 lines) and Wnt3a-producing cells (CRL-2647 cells). Calvin Kuo (Palo Alto, CA, USA) kindly provided R-spondin-producing cells. Gijs van den Brink (University of Amsterdam, Amsterdam, the Netherlands) kindly provided Noggin-producing cells. Complete media with growth factors (CMGF^+^) and without growth factors (CMGF^–^), differentiation media, and 3 conditioned media (Wnt3a, R-spondin, and Noggin) were prepared as reported previously (*13**,**16**,**17*).

We grew jejunal HIE cultures (J2 or J3 lines) as undifferentiated 3D cultures in the presence of CMGF^+^ supplemented with 10 μmol/L Y-27632, as described previously (*17*). After 7 days, highly dense 3D cultures were either split 1:2, frozen in LN_2_, or dissociated into a single cell suspension and plated as undifferentiated monolayers. After culture for 24 h at 37°C in 5% CO_2_, we replaced CMGF^+^ supplemented with 10 μmol/L Y-27632 with differentiation medium to induce monolayer differentiation.

### Infection Experiments and Viral Replication

We performed all infections in triplicate on 100% confluent 4-day-old differentiated HIE (J3 line) monolayers, except when specified that the J2 line was used. In some experiments, we pretreated monolayers with 1% sow bile included in the differentiation medium 48 h before infection. In other experiments, we differentiated HIE monolayers without pretreatment, and infected them in the presence of 500 μmol/L of glycochenodeoxycholic acid (GCDCA; Sigma, St. Louis, MO, USA) or with 500 μM GCDCA plus 50 μM ceramide.

To determine viral infectivity, we inoculated duplicate 96-well plates with 100 μL of fecal filtrate (Technical Appendix) at 1:10, 1:100, and 1:1000 dilution. After 1 h incubation at 37°C in 5% CO_2,_ we washed the monolayers twice with CMGF^–^ and added 100 μL of differentiation medium containing 1% sow bile, 500 μmol/L GCDCA, or 500 μM GCDCA plus 50 μmol/L ceramide to each well. For each set of infections, we immediately froze 1 plate at −70°C and incubated a duplicate plate at 37°C in 5% CO_2_ for 72 h and froze it at −70°C. We used quantitative reverse transcription real-time PCR to determine the amount of norovirus RNA from input virus and from HIE monolayers at 1 hour postinfection (hpi) and at 72 hpi. Standard curve based on quantified RNA transcripts was included.

### Inactivation Treatments

#### Alcohol Treatment

We diluted 10% fecal filtrates 1:10 in 70% ethanol or isopropanol solutions and incubated them for 1 min or 5 min. We then neutralized the alcohols in the samples by adding 9 volumes of CMGF supplemented with 10% fetal bovine serum (FBS). We included a nontreatment control and an alcohol neutralization control in each experiment.

### Chlorine Suspension Assays

We prepared fresh chlorine stock solutions at 1,000 ppm and 10,000 ppm by diluting commercial bleach (6% sodium hypochlorite) in cell culture–grade water. We diluted 20 μL of 10% fecal filtrates in an appropriate volume of chlorine stock solutions to achieve a series of total chlorine concentrations of 5–5000 ppm. After incubating the solutions for 1 min at room temperature, we added sodium thiosulfate (final concentration 50 mg/L) to neutralize free chlorine. We included a nontreatment control and a chlorine neutralization control in each experiment.

## Results

### HIE Model and Small Intestine Complexity

**Figure 1:**
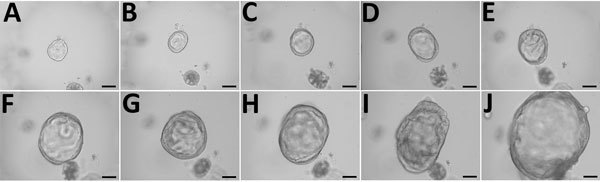
Growth of 3-dimensional human intestinal enteroid (HIE) embedded in Matrigel in the presence of complete media with growth factors containing Wnt3a, R-spondin, and Noggin as part of a study of human norovirus replication in HIEs. Microscopy images show the growth of a representative undifferentiated HIE: A) day 1; B) day 2; C) day 3; D) day 4; E) day 5; F) day 6; G) day 7; H) day 8; I) day 9; J) day 10. Scale bars indicate 100 μm.

**Figure 2:**
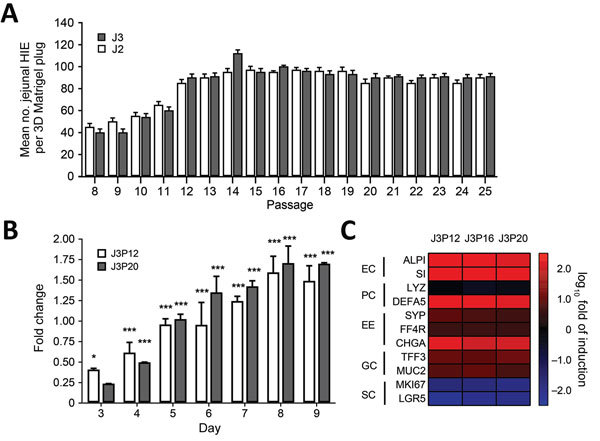
Characterization of differentiated and undifferentiated HIE in a study of human norovirus replication in HIEs. A) Quantification of undifferentiated HIE generated on each passage. Undifferentiated HIEs derived from 2 donors (J2P7 and J3P7) frozen at passage 7 (P7) were recovered from LN_2_ and embedded in Matrigel (BD Biosciences, San Jose, CA, USA) (4 plugs per HIE). Cell count was performed at day 7. On that day, undifferentiated HIEs were split 1:2 or 1:3, depending on density, and seeded again in Matrigel. All available wells (n > 4) per passage were counted. Error bars indicate SD. B) Analysis of stem cell proliferation marker gene LGR5 expression by quantitative reverse transcription PCR in undifferentiated HIEs. HIEs were embedded into Matrigel, seeded in individual wells, and cultured in the presence of complete media with growth factors. RNA was isolated from 2 wells at 1 hour postseed (day 0) and each day during days 3–9. LGR5 expression was normalized to GAPDH and expressed as fold change relative to day 0 (n = 2 wells/bar). Two different passages were assayed (P12 and P20). Error bars indicate SDs; asterisks indicate significant difference from day 0: *p<0.05; ***p<0.001. C) Heat map based on log (2^–∆∆Ct^) comparing gene expression levels for markers of differentiated small intestinal epithelial cells between undifferentiated and 4-day differentiated HIE monolayers. Experiments were performed with 3 independent cell passages (P12, P16, and P20). Transcripts were normalized to GAPDH levels. Shown are markers for enterocytes (EC), Paneth cells (PC), enteroendocrine cells (EE), goblet cells (GC), and stem cells (SC). Gene symbols: ALPI, intestinal-type alkaline phosphatase; CHGA, chromogranin A; DEFA5, defensin α 5; FFA4R, free fatty acid receptor 4; GAPDH, glyceraldehyde-3-phosphate dehydrogenase; LGR5, leucin-rich repeat-containing G-protein-couple receptor 5; LYZ, lysosyme; MKI67, marker of proliferation Ki-67; MUC2, mucin 2; SI, sucrose isomaltase; SYP, synaptophysin; TFF3, trefoil factor 3. 3D, 3-dimensional; HIE, human intestinal enteroid.

We recovered jejunal HIE cultures from 2 donors (J2 and J3) frozen at passage 7 (P7) from LN_2_ and grew them as 3D cultures in Matrigel (BD Biosciences, San Jose, CA, USA). Within 24 hours, cells formed small cystic or multilobular HIEs and continued to grow in the presence of CMGF^+^ medium (Figure 1). With each passage, the number of 3D HIEs doubled, reaching a maximum Matrigel capacity of 100/plug. We were able to culture HIEs for >4 months (16−17 consecutive passages) (Figure 2, panel A). We confirmed that the number of highly proliferative stem cells increased over time, as shown by enhanced transcriptional levels of LGR5^+^ (Figure 2, panel B), whereas differentiated monolayers’ LGR5^+^ expression levels were greatly reduced and lineage-specific markers (SI, ALPI, TFF3, MUC2, FFA4R) were increased (Figure 2, panel C).

### Human Norovirus Infection of Jejunal HIEs

**Figure 3:**
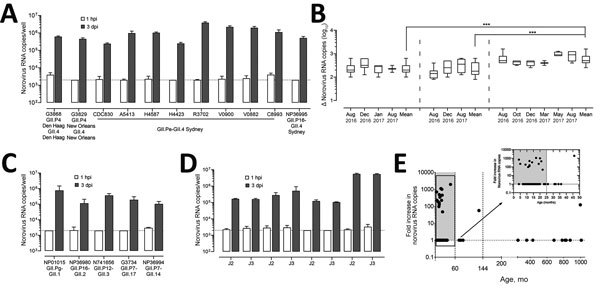
Evaluation of human norovirus replication in human intestinal enteroids (HIEs). A) Jejunal HIE monolayers (J3 line) inoculated with GII.4 P4 Den Haag-GII.4 Den Haag (6.2 × 10^4^ RNA copies/well), GII.4 P4 New Orleans-GII.4 New Orleans (5.3 × 10^5^ RNA copies/ well), GII.4 P16-GII.4 Sydney (1.5 × 10^6^ RNA copies/well) or GII.4 Pe-GII.4 Sydney (1.8 × 10^5^ to 3.0 × 10^6^ RNA copies/well). Each experiment was performed 3 times with 3 technical replicates each. B) Replicated infections with fecal filtrates. GII.4 P4 Den Haag-GII.4 Den Haag (6.2 × 10^4^ RNA copies/well), GII.4 P4 New Orleans-GII.4 New Orleans (5.3 × 10^5^ RNA copies/well), GII.4 Pe-GII.4 Sydney (1.8 × 10^5^ RNA copies/well) were included as positive controls throughout 1 year to assess the reproducibility of the HIE culture system. Data are shown as log increase in norovirus RNA per well at 3 dpi compared with 1 hpi. Boxes represent 25th percentile, median, and 75th percentile, and whiskers show minimum and maximum values. Each box represents all experiments performed during the indicated month (GII.P4 New Orleans-GII.4 New Orleans, n = 4–8, total: 18; GII.P4 Den Haag-GII.4 Den Haag, n = 6–15, total: 33; GII.Pe-GII.4 Sydney, n = 6–20, total: 35). ***p<0.0001 comparing log increase in norovirus RNA between 2 GII.4 variants. C) Jejunal HIE monolayers inoculated with NP01015 (GII.Pg-GII.1) at 4.8 × 10^6^ RNA copies per well, NP36980 (GII.P16-GII.2) at 1.51 × 10^6^ RNA copies/well, N741656 (GII.P12-GII.3) at 1.5 × 10^6^ RNA copies/well, G3734 (GII.P7-GII.17) at 2.0 × 10^3^ RNA copies/well or NP36994 (GII.P7–GII.14) at 5.9 × 10^6^ RNA copies/well. Data represent mean ± SD of 3 experiments with 3 technical replicates for each experiment. D) Jejunal HIE monolayers (lines J2 and J3) inoculated with G3734 (GII.P7-GII.17) at 2.0 × 10^3^ RNA copies/well, N741656 (GII.P12-GII.3) at 1.5 × 10^6^ RNA copies/well, NP36980 (GII.P16-GII.2) at 1.51 × 10^6^ RNA copies/well, or R3702 (GII.P16-GII.4 Sydney) at 7.7 × 10^6^ RNA copies/well. Data represent mean ± SD of 2 experiments with 3 technical replicates for each experiment. E) Relationship between age of patient from whom fecal sample was collected and success of replication in HIE. We collected 80 fecal samples during 2000–2017 from children <12 years of age (n+62) and from adults (n=18). No replication is indicated as 1. For panels A, C, and D, dotted lines represent quantitative RT-qPCR limit of detection. dpi, days postinfection; hpi, hours postinfection.

A previous study demonstrated the replication of GII.4 norovirus in HIEs (*16*). To evaluate whether those results could be reproduced, we infected jejunal HIEs (line J3) with GII.4 fecal filtrates (GII.P4 Den Haag-GII.4 Den Haag, GII.P4 New Orleans-GII.4 New Orleans, GII.Pe-GII.4 Sydney, and GII.P16-GII.4 Sydney). At 72 hpi, we detected 100- to 1,100-fold increases in viral RNA copies per well for all GII.4 fecal filtrates compared with viral RNA levels detected at 1 hpi (Figure 3, panel A; Table 2).

**Table 2:** Norovirus fecal filtrates successfully cultivated in study of human norovirus replication in human intestinal enteroids

Sample ID	Genotype, RdRp-Capsid*	Mean norovirus RNA copies/μL	Participant age, mo	Collection date†	Storage condition‡	Fold virus RNA increase, log_10_§	
NP01015	GII.Pg-GII.1	1.4 × 10^5^	4	2017 Mar	−70°C	696 (2.8)	
NP36980	GII.P16-GII.2	1.9 × 10^3^	132	2017 Feb	−70°C	56 (1.8)	
N741656	GII.P12-GII.3	1.4 × 10^5^	9	2012 Aug	−70°C	181 (2.3)	
G3868	GII.P4 Den Haag-GII.4 Den Haag	2.0 × 10^5^	12	2015 May	−70°C	155 (2.2)	
G3829	GII.P4 New Orleans-GII.4 New Orleans	4.1 × 10^5^	5	2013 Apr	−70°C	227 (2.4)	
A5413	GII.Pe-GII.4 Sydney	1.6 × 10^6^	12	2012	−70°C	487 (2.7)	
R3702	GII.Pe-GII.4 Sydney	7.7 × 10^6^	46	2015 Jan	−70°C	1236 (3.0)	
V0882	GII.Pe-GII.4 Sydney	4.0 × 10^6^	20	2015 Feb	−70°C	998 (3.0)	
V0900	GII.Pe-GII.4 Sydney	5.1 × 10^5^	17	2015 Feb	−70°C	1102 (3.0)	
H4423	GII.Pe-GII.4 Sydney	5.9 × 10^5^	12	2015 Feb	−70°C	125 (2.1)	
H4587	GII.Pe-GII.4 Sydney	4.4 × 10^6^	21	2015 Apr	−70°C	448 (2.7)	
C8993	GII.Pe-GII.4 Sydney	1.7 × 10^7^	19	2015 Mar	−70°C	223 (2.3)	
CDC830¶	GII.Pe-GII.4 Sydney	2.9 × 10^6^	996	2016 Apr	4°C	121 (2.1)	
NP36995	GII.P16-GII.4 Sydney	2.1 × 10^6^	14	2016 Dec	−70°C	340 (2.5)	
NP36994	GII.P7-GII.14	6.1 × 10^4^	22	2016 Dec	−70°C		47 (1.6)
G3734	GII.Pe-GII.17	3.4 × 10^5^	11	2010 Oct	−70°C	96 (1.9)	

*Dual genotyping based on sequencing partial RdRp and capsid regions (*26**,**27*). RdRp, RNA-dependent RNA polymerase. †When month was not available, only year of collection is reported. ‡Samples were kept from collection time until testing at the indicated temperature. §Virus RNA increase after 3 days postinfection expressed as fold increase and log_10_ fold increase. For each fecal filtrate, data represent mean viral RNA increase from 3 experiments with 3 technical replicates each. ¶CDC830 was stored at 4°C for 2 mo before cultivation.

To further evaluate the reproducibility of the system, we included GII.4 fecal filtrates (GII.4 Den Haag, GII.4 New Orleans, and GII.4 Sydney) in each infection experiment conducted during August 2016–2017. We observed consistent replication of the 3 strains without significant differences in viral titers (Figure 3, panel B). The mean log_10_ increase was 2.7 (95% CI 2.68–2.82; n = 35) for GII.4 Sydney, 2.4 (95% CI 2.29–2.45; n = 33) for GII.4 Den Haag, and 2.3 (95% CI 2.17–2.46; n = 18) for GII.4 New Orleans. We observed significantly higher-fold increases on viral RNA titers for GII.4 Sydney compared with GII.4 Den Haag and GII.4 New Orleans (p<0.0001).

To evaluate whether HIEs support replication of non-GII.4 strains, we inoculated monolayers with different GI-, GII-, and GIV-positive fecal filtrates (Table 1). We observed viral replication for GII.Pg-GII.1 (2.6 log_10_), GII.P16-GII.2 (2.8 log_10_), GII.P12-GII.3 (2.3 log_10_), GII.P7-GII.14 (1.7 log_10_), and GII.Pe-GII.17 (1.9 log_10_) strains (Table 2; Figure 3, panel C). We did not observe replication of GI, GIV, and other GII genotypes. We also confirmed that both J2 and J3 HIE lines support human norovirus replication without significant differences in -fold change between the 2 cell lines (Figure 3, panel D). Most (15/16; 94%) samples that successfully replicated had been stored at −70°C (Table 2) and were collected from children <2 years of age (13/16; 81%) (Figure 3, panel E). CDC830 was stored at 4°C for 2 months before cultivation; this sample was collected from an adult 83 years of age.

**Figure 4:**
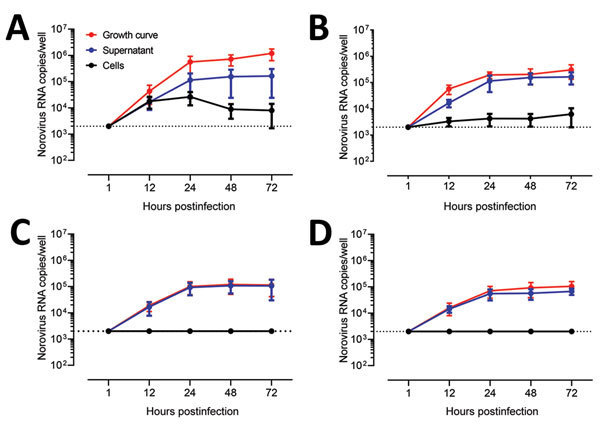
Evaluation as human norovirus replication in human intestinal enteroids (HIEs) by assessment of kinetics of infection for 4 GII genotypes. We inoculated jejunal HIE monolayers (J3 line) with A) GII.4 Pe-GII.4 Sydney (3.3 × 10^5^ RNA copies/well), B) GII.P12-GII.3 (5.3 × 10^5^ RNA copies/well), C) GII.P16-GII.2 (3.2 × 10^5^ RNA copies/well), or D) GII.Pg-GII.1 (9.3 × 10^5^ RNA copies/well). After 1 h at 37°C in 5% CO_2_, monolayers were washed, and media was replaced with differentiation media and incubated for 3 d. For the growth curve, we extracted RNA from frozen lysates (cells and supernatant) at the indicated time points. For the cells vs. supernatant experiment, we removed supernatants by centrifugation before harvesting the cells. Data represent mean ± SD of 2 experiments with 3 wells for each time point. Dotted lines represent RT-quantitative PCR limit of detection.

We next evaluated replication of human norovirus in HIEs by assessing the kinetics of infection for 4 GII genotypes (GII.1, GII.2, GII.3, and GII.4 Sydney). Consistent with a successful infection, norovirus RNA levels increased at 12 hpi, reaching a plateau at 24 hpi; no significant further increase at 72 hpi was observed for any of the genotypes (Figure 4). Despite a similar viral input level (3.3–9.3 × 10^5^ copies/well), GII.4 Sydney infected HIEs with higher efficiency than did the other 3 genotypes (Figure 4), as shown by higher levels of viral RNA in cells and supernatant.

**Figure 5:**
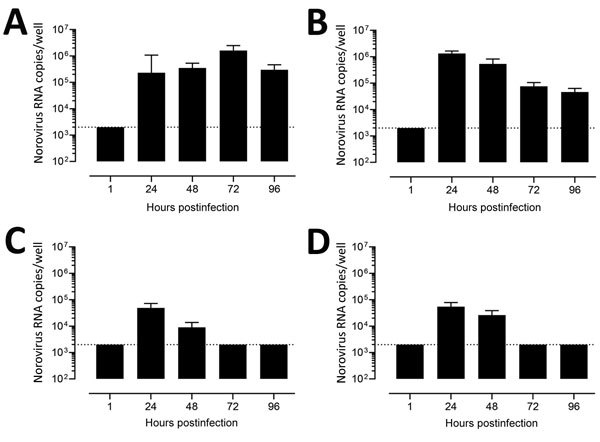
Confirmatory testing for human norovirus replication in human intestinal enteroids (HIEs). We inoculated jejunal HIE monolayers (J3 line) with A) GII.4 Pe-GII.4 Sydney (3.3 × 10^5^ RNA copies/well), B) GII.P12-GII.3 (5.3 × 10^5^ RNA copies/well), C) GII.P16-GII.2 (3.2 × 10^5^ RNA copies/well), or D) GII.Pg-GII.1 (9.3 × 10^5^ RNA copies/well). After 1 h at 37°C in 5% CO_2_, monolayers were washed, and media was replaced with differentiation medium and incubated at 37°C and 5% CO_2_. At 24, 48, and 72 hours postinfection, we removed the medium and added fresh differentiation media. At 96 hours postinfection, we removed the medium. We extracted RNA and quantified it by quantitative reverse transcription PCR from the supernatant at each time point. Data represent mean ± SD of 2 experiments with 3 wells for each time point. Dotted lines represent RT-qPCR limit of detection.

To further confirm the production and release of norovirus from cells, we quantified viral titers in supernatants collected from cell cultures and replaced the differentiation media every 24 hours after infection (Figure 5). We detected viral RNA in supernatants collected at 24 hpi and 48 hpi for all infections. Higher and more consistent levels of norovirus RNA were detected in HIE infected with GII.4 Sydney up to 96 hpi, whereas the initial RNA levels detected for GII.1, GII.2, and GII.3 declined and became undetectable after 48 hpi for GII.1 and GII.2. These data clearly demonstrate that, although HIEs are permissive to infection with different norovirus genotypes, GII.3 and GII.4 replicated with higher efficiency.

**Figure 6:**
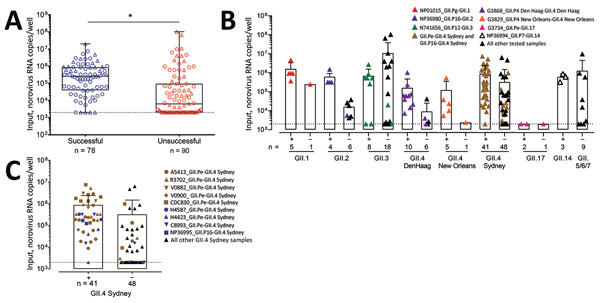
Comparison of amount of input viral RNA with success of human norovirus replication in human intestinal enteroids (HIEs). A) We infected HIE monolayers with undiluted or prediluted (1:10; 1:100; 1:1000) 10% fecal filtrates. Each dot represents the input norovirus RNA per well of a single experiment (n = 168) that resulted in successful (n = 78) or unsuccessful (n = 90) virus replication. Boxes represent 25th percentile, median, and 75th percentile, and whiskers show the minimum and maximum values. Circles indicate GII.4 genotypes and triangles non-GII.4 genotypes. ***p<0.001 by Mann-Whitney test. B, C) Role of initial norovirus RNA input in successful (+) and unsuccessful (–) human norovirus infections. We infected HIE monolayers with undiluted or prediluted (1:10; 1:100; 1:1000) 10% fecal filtrates and incubated them at 37°C in 5% CO_2_ for 3 d. We extracted RNA and quantified it by quantitative reverse transcription PCR from frozen lysates (cells and supernatant) at 1 hour postinfection and 3 days postinfection. Data points represent individual experiments. Bars represent mean + SD. Dotted lines represent RT-qPCR limit of detection. Samples that successfully replicate at high, but not low, concentration are colored and listed in Table 2.

**Figure 7:**
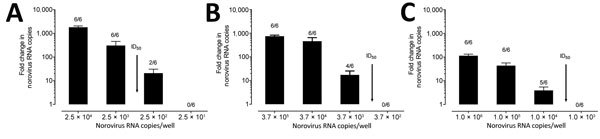
Determination of ID_50_ required for human norovirus replication in human intestinal enteroids (HIEs). We inoculated HIE monolayers in triplicate with 10-fold serial diluted fecal filtrates A) A5413_GII.4 Sydney, B) G3868_GII.4 Den Haag, or C) N741656 _GII.3 RNA copies and incubated them for 1 h at 37°C. We washed the monolayers 3 times and cultured them in differentiation media for 3 d. We extracted RNA and quantified it by quantitative reverse transcription PCR from frozen lysates (cells and supernatant) at 1 hour postinfection and 3 days postinfection. We calculated ID_50_ using the Reed-Muench method (*28*). ID_50_, 50% infectious dose.

We also compared the amount of input viral RNA with the success of replication. Samples that replicated successfully had a significantly higher input titer compared with strains that did not replicate (p<0.0001) (Figure 6, panel A). Stratified by genotype, the effect of the initial input amount of virus was observed for infections performed with GII.1, GII.2, GII.4 Den Haag, GII.4 New Orleans, and GII.4 Sydney viruses (Figure 6, panels B, C). To further confirm the role of the amount of virus inoculum on the success of replication, we infected HIE monolayers with 10-fold serial dilutions of GII.3 and GII.4 fecal filtrates. The dose required to produce infection in 50% of the inoculated wells (ID_50_) was 2.1 × 10^3^ genome copies/well for GII.4 Den Haag, 4.4 × 10^2^ genome copies/well for GII.4 Sydney, and 4.0 × 10^3^ genome copies/well for GII.3, based on the Reed-Muench method (Figure 7) (*28*).

### Inactivation of Human Norovirus by Alcohols

**Figure 8:**
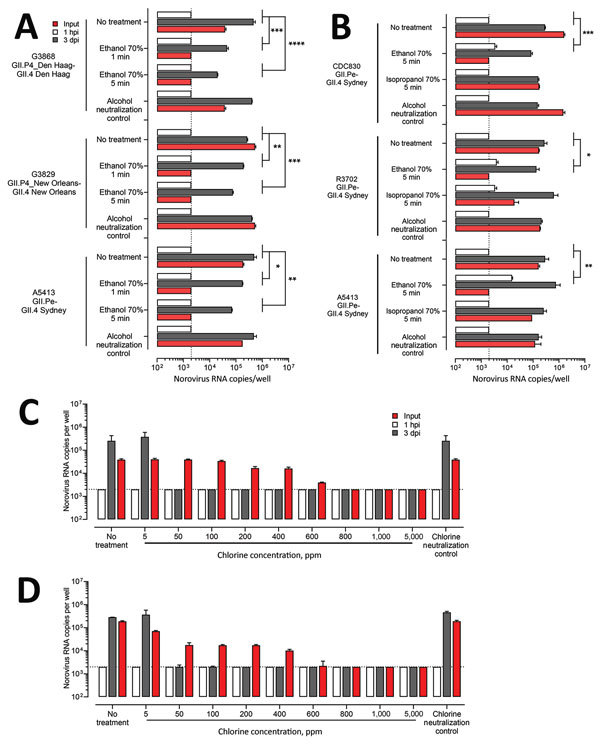
Inactivation of human norovirus with 70% alcohol or chlorine solution in suspension. A) Ten percent fecal filtrates (G3868 [GII.4 Den Haag], 2.04 × 10^6^ RNA copies; G3829 [GII.4 New Orleans], 4.14 × 10^6^ RNA copies; A5413 [GII.4 Sydney], 1.58 × 10^7^ RNA copies) were either treated or not treated with 70% ethanol for 1 min or 5 min at room temperature. We added complete media without growth factors supplemented with 10% fetal bovine serum to neutralize remaining ethanol. B) Ten percent fecal filtrates from 3 GII.4 Sydney strains (CDC830, 2.89 × 10^7^ RNA copies; R3702, 7.73 × 10^7^ RNA copies; A5413, 1.58 × 10^7^ RNA copies) were either treated or not treated with 70% ethanol or 70% isopropanol for 5 min at room temperature and neutralized with complete media without growth factors supplemented with 10% fetal bovine serum. C, D) Ten percent fecal filtrates (G3868, 2.04 × 10^6^ RNA copies [C]; A5413, 1.58 × 10^7^ RNA copies [D]) were either treated or not treated with freshly prepared chlorine solutions of increasing concentrations (5, 50, 100, 200, 400, 600, 800, 1,000, and 5,000 ppm) for 1 min at room temperature. Sodium thiosulfate (final concentration 50 mg/L) was added to neutralize the remaining free chlorine. For all experiments, HIEs were then infected with 100 μL of treated or not treated fecal filtrate. After 1 h at 37°C in 5% CO_2_, we washed the monolayers, added differentiation media, and incubated for 3 d. Data represent mean ± SD of 2 experiments with 3 wells for each treatment and time point. For each fecal filtrate, we performed 1-way analysis of variance followed by Dunnett’s test. Dotted lines represent RT-qPCR limit of detection. p values are compared with the nontreated fecal filtrate: *p<0.05; **p<0.01; ***p<0.001; ****p<0.0001. dpi, days postinfection; hpi, hours postinfection.

We next evaluated the efficacy of alcohols to inactivate infectious human norovirus by using 3 successfully replicating GII.4 viruses (GII.4 Den Haag, GII.4 New Orleans, and GII.4 Sydney). Although replication levels of fecal filtrates exposed to 70% ethanol for 1 and 5 minutes were significantly lower compared with nontreated fecal filtrates (p<0.05), none of the GII.4 viruses was completely inactivated by ethanol (Figure 8, panel A). In addition, we treated 2 GII.4 Sydney fecal filtrates (R3702 and CDC830) with 70% ethanol or 70% isopropanol for 5 minutes to rule out the possibility that the observed inactivation patterns were sample specific (Figure 8, panel B). We observed no complete inactivation for any of the tested samples, although the replication levels after treatment with 70% ethanol were up to 0.7 log_10_ lower, and we found no reduction after exposure to isopropanol (Figure 8, panel B). Following treatment of the fecal filtrates with 70% isopropanol for 5 minutes, norovirus input RNA was still detectable, whereas input titers after 70% alcohol treatment were reduced (1.3 to 2.9 log_10_).

### Inactivation of Human Norovirus by Chlorine

To evaluate the ability of chlorine to effectively inactivate infectious human norovirus, we treated fecal filtrates of 3 GII.4 viruses with increasing concentrations of chlorine (5–5,000 ppm) for 1 minute. Compared with nontreated controls, all chlorine concentrations >50 ppm completely inactivated GII.4 Den Haag, GII.4 Sydney (Figure 8, panels C, D), and GII.4 New Orleans (data not shown). Norovirus input RNA was detectable in all samples that were treated with <600 ppm of chlorine.

## Discussion

Since the discovery of Norwalk virus, many attempts have been made to culture human noroviruses; most efforts were unsuccessful, or the results were not reproducible in other laboratories (*2**–**6**,**9**,**10*). The successful long-term expansion of intestinal epithelial organoids has been a major breakthrough in the field of in vitro culture of intestinal epithelium (*12**,**14**,**15*). Recent studies show that HIEs support replication of human norovirus and other enteric viruses (*13**,**16**,**29*) and enable analysis of the innate immune response against these viruses (*30*). In this study, we showed successful replication of 6 GII norovirus genotypes (GII.1, GII.2, GII.3, GII.4, GII.14, and GII.17), including 3 GII.4 variants. Repeated infections conducted over a 1-year period showed consistent increase in viral titers of these 3 GII.4 variant strains, demonstrating that the HIE model is robust. Our data also demonstrate that, after initial confirmation of infectivity, storage of fecal samples at −70°C will preserve virus infectivity for at least 1 year.

We showed successful replication for 6 of the 14 genotypes tested in this study, although the success rate varied. Strain-specific differences have been reported for other viruses grown in HIEs (*13**,**29*). For example, compared with echovirus 11 and coxsackievirus B, enterovirus 71 replicates to significantly lower levels in HIEs (*29*). Enteroids also support robust replication of human rotavirus strains Ito (G3P[8]) and Wa (G1P[8]) but not the attenuated G1P[8] human rotavirus vaccine strain (*13*). Ettayebi et al. also demonstrated that GII.4 Sydney strains infect enteroids with higher efficiency than do GI.1, GII.3, and GII.17 viruses (*16*); in our study, GII.4 norovirus strains replicated at higher efficiency than did GII.1, GII.2, and GII.3 viruses. In addition, the ID_50_ values for GII.4 and GII.3 viruses were similar but slightly lower (2–5×) than reported previously (*16*). Taken together, these results indicate that high viral RNA titers are not a guarantee for suc-cessful replication, perhaps suggesting that fecal specimens that do show norovirus replication may contain large num-bers of noninfectious particles.

Until now, evaluation of control measures for human norovirus, including disinfection measures, has relied primarily on the use of cultivable surrogate viruses (*24*). Although these viruses are similar in size and genome organization, none completely mimics the inactivation patterns of human norovirus based on reduction of viral RNA titers. We demonstrated that the HIE model can be used to evaluate the effectiveness of alcohols and chlorine against human norovirus. Although 5 minutes of exposure to 70% ethanol and isopropanol slightly reduced viral RNA levels, overall, the alcohols did not inactivate GII.4 viruses. These results are in agreement with a previous study that, based on lack of reduction of viral RNA titers, suggested that GII human noroviruses are not affected by alcohol (*24*). In a comprehensive study comparing different cultivable surrogate viruses for human norovirus, Cromeans et al. (*24*) showed that Tulane virus, but not feline calcivirus or MNV, was resistant to alcohols. Using HIEs, we now demonstrate that human norovirus closely resembles Tulane virus when measuring inactivation by alcohols.

For chlorine, our data showed that complete inactivation of 3 different GII.4 strains could be achieved with concentrations as low as 50 ppm. These results are consistent with a recent report indicating that treatment with chlorine concentrations <50 ppm were not sufficient to inactivate human norovirus in secondary effluents from water treatment plants (*31*). In conclusion, our inactivation data demonstrate that chlorine, but not alcohol, completely inactivates human norovirus and that evaluation of inactivation strategies based only on detection of viral RNA does not always reflect the effectiveness of the treatment.

Our study has several limitations. First, the success rate of samples with a moderate viral RNA titer was relatively low, and thus far we have had no success with GI and GIV samples. However, because we were also not able to replicate several high viral load GII samples, other, currently unknown, factors also contribute to successful replication. Second, although we demonstrated that infectious particles are produced and we were able to measure complete inactivation by chlorine treatment, we analyzed only viral RNA levels, not protein levels. Further work is needed on the amount of chlorine required to inactivate human norovirus because we used fecal samples, which inherently have a high chlorine burden, and we measured total chlorine, whereas the level of free chlorine is what actually determines inactivation. Finally, the HIE model is costly and labor intensive; additional improvements are required to make it more affordable and widely available.

In conclusion, we confirmed that the HIE system to culture human norovirus (*16*) can be successfully implemented in another laboratory. The culture system supported identical levels of replication of a panel of human norovirus strains consistently for >1 year. The success of replication depends on genotype and initial virus titer but also on other unknown factors. Additional HIE cell lines need to be tested or cultures need to be enriched for enterocytes (*32*) because replication of some noroviruses is restricted by cell line characteristics (*16*). In addition, whether infectivity is limited by the presence of virus-specific fecal antibodies and the possibility that cellular host factors may prevent or limit replication of certain genotypes all indicate that more research is needed to further optimize this cultivation system.

Technical Appendix Detailed description of materials and methods for experiments involving human norovirus replication in human intestinal enteroids.
