# Supplementary material for: Human Norovirus Replication in Human Intestinal Enteroids as Model to Evaluate Virus Inactivation
Source: Emerg Infect Dis. 2018 Aug;24(8):1453–64. doi: 10.3201/eid2408.180126 (PMC6056096; doi:10.3201/eid2408.180126)
Supplement: Technical Appendix — Detailed description of materials and methods for experiments involving human norovirus replication in human intestinal enteroids. [file 18-0126-Techapp-s1.pdf]

Article DOI: https://doi.org/10.3201/eid2408.180126

Human Norovirus Replication in Human Intestinal Enteroids as Model to Evaluate Virus Inactivation

Technical Appendix

Materials and Methods

Fecal Samples

This investigation was determined by CDC to be public health non-research, and therefore was not subject to institutional review board review. We included 80 fecal samples in the study (Technical Appendix Table). We collected the samples during 2000–2017 from children ≤12 years old (n = 62) and adults (n = 18). We collected 67 samples from sporadic cases and 13 samples from norovirus outbreaks. This collection included 12 genogroup (G) I, 65 GII, and 3 GIV human norovirus positive fecal samples. We stored the samples at the indicated temperature from collection time until the time of testing. We prepared 10% fecal suspensions by adding 0.5 g of whole fecal sample or 500 μL of liquid fecal sample to 4.5 mL of PBS. We vortexed the fecal suspensions for 30 seconds, kept them at room temperature for 5 minutes, and vortexed them again. We sonicated the samples 3 times for 1 minute at the highest setting, with 1-minute periods on ice after each sonication step. We removed the solids by centrifugation for 10 minutes at 10,000 × g and serially filtered the supernatants through 5 μm, 1 μm, 0.45 μm, and 0.22 μm filters. Aliquots of the resulting 10% clarified fecal filtrate were stored at −70°C.

Ten-fold serial dilutions of the 10% fecal filtrate (until viral RNA input was undetectable by real-time RT-PCR) were tested in triplicate. All samples were tested during April 2016–December 2017.

Human Intestinal Enteroid Culture and Media

Secretor-positive jejunal HIE cultures (J2 and J3 lines) and Wnt3a-producing cells (CRL-2647 cells) were provided by Baylor College of Medicine. R-spondin-producing cells were

kindly provided by Dr. Calvin Kuo, Palo Alto, CA. Noggin-producing cells were kindly provided by Dr. Gijs van den Brink, University of Amsterdam, Netherlands. Complete media with and without growth factors ($CMGF^+$ and $CMGF^–$, respectively), differentiation media, and Wnt3a- R-spondin- and Noggin -conditioned media were prepared as reported previously (*1*,*2*).

Jejunal HIE cultures (J2 or J3 lines) were grown as undifferentiated 3-dimensional (3D) cultures, as described previously (*2*) with minor modifications. Briefly, HIEs were recovered from liquid nitrogen (LN_2), suspended in 20 μL of Matrigel® (≈40 crypts), plated in a single well of a 24-well plate, and grown as 3D cultures in $CMGF^+$ medium supplemented with 10 μM Y-27632 (Sigma). We replaced the medium every 48 hours. After 7 days, highly dense 3D cultures were either split 1:2 and embedded in Matrigel, frozen in liquid nitrogen for further use, or dissociated into single cell suspension and plated as undifferentiated monolayers, as described previously (*2*) (Technical Appendix Figure 1).

For all infections, we washed undifferentiated HIEs with 0.5 mM EDTA in ice-cold PBS (without calcium chloride–magnesium chloride) and dissociated into single cell suspension with 0.05% trypsin/0.5 mM EDTA. We seeded 96 well plates with $1–2 \times 10^5$ cells/well to form monolayers, as described previously (*2*). After 24 hours, $CMGF^+$ supplemented with 10 μM Y-27632 was replaced with differentiation medium, which was refreshed every 48 hours during 4 days.

Total RNA Isolation and Gene Expression Analysis

We isolated total RNA from differentiated and undifferentiated HIE monolayers using MagMAX-96 Total RNA Isolation Kit according to the manufacturer’s instructions. For each sample, 1 μg of RNA was reverse transcribed using the High Capacity RNA to cDNA kit (Applied Biosystems, Foster City, CA, USA). We performed real-time PCR amplification using TaqMan Fast Advance master mix and TaqMan Gene Expression assays (Life Technologies, Grand Island, NY, USA) or IDT PrimeTime qPCR Assays (Technical Appendix Table 1) in an Applied Biosystems 7500 platform. Expression levels were normalized to GAPDH and fold-change of expression level was calculated using the comparative Ct method ($2^{-\Delta\Delta Ct}$), as described previously (*3*). Heat maps were created using GraphPad Prism7.0 (GraphPad Software, La Jolla, CA, USA).

Infection Experiments and Viral Replication

We performed all infections in triplicate on 100% confluent 4-day-old differentiated HIE (J3 line) monolayers in 96-well plates, except when specified that J2 line was used. In some experiments, monolayers were pretreated with 1% sow bile included in the differentiation medium 48 hours before infection. In other experiments, HIE monolayers were differentiated 4 days before infection without pretreatment, and infected in the presence of 500 μM of glycochenodeoxycholic acid (GCDCA; Sigma) or 500 μM GCDCA plus 50 μM of a ceramide. Ten percent fecal filtrates were pre-diluted 1:10, 1:100, 1:1000, and 1:10000 in PBS. Each dilution was further diluted 1:20 in $CMGF^{-}$ with 1% sow bile, 500 μM GCDCA, or 500 μM GCDCA plus 50 μM of a ceramide. In all experiments, monolayers were washed twice with $CMGF^{-}$ and inoculated with 100 μL of each fecal filtrate (Technical Appendix Figure 2). Bile was collected from sows and piglets under a study protocol approved by the Animal Care and Use Committee of Baylor College of Medicine and was conducted in accordance with the Guide for the Care and Use of Laboratory Animals (*4*).

To determine viral infectivity, we inoculated duplicate plates. After 1 hour of incubation at 37°C and 5% CO_2, we washed the monolayers twice with $CMGF^{-}$ and added 100 μL of differentiation medium containing 1% sow bile, 500 μM GCDCA, or 500 μM GCDCA plus 50 μM ceramide to each well. For each set of infections, 1 plate was immediately frozen at −70°C and a duplicate plate was incubated at 37°C, 5% CO_2 for 72 hours and frozen at −70°C. Viral RNA was extracted from input virus, and HIE monolayers at 1 hour postinfection (hpi) and 3 days postinfection (dpi) were determined by RT-qPCR as described below (Technical Appendix Figure 2).

Norovirus Detection, Quantification, and Genotyping

We extracted viral RNA from input virus and HIE monolayers at 1 hpi and 3 dpi using the KingFisher instrument and MagMAX–96 Viral RNA Isolation Kit (Ambion, Austin, TX, USA) according to the manufacturer’s instructions. For infected cells, we performed RNA extraction with minor modifications. Briefly, we added 250 μL of lysis buffer directly to each well and incubated them for 10 minutes at room temperature. We then transferred the cell lysates to a processing plate for RNA extraction according to the manufacturer’s instructions. Norovirus RNA was detected by GI/GII TaqMan real-time RT-PCR (RT-qPCR) (*5*). We generated standard

curves using 10-fold serial dilutions of GI.4, GII.4, and GIV RNA transcripts. The real-time RT-PCR limit of detection was 200 RNA copies per 5 μL of RNA (or 4.0×10^3 RNA copies/well). Samples with titer below the limit of detection were arbitrarily assigned to half of the limit of detection (100 RNA copies per 5 μL of RNA or 2.0×10^3 RNA copies/well). Positive samples were genotyped by a dual typing RT-PCR using oligonucleotide primer sets specific for GI and GII viruses (*5*). PCR products were visualized on a 2% agarose gel and purified by ExoSAP-IT (Affymetrix, Cleveland, OH, USA). Genotypes were assigned by phylogenetic analysis using the unweighted-pair group method with reference sequences used by CaliciNet (*6*) for capsid typing, or the Norovirus Typing Tool, version 2.0 (*7,8*).

Virus Growth Kinetics

For growth curves, cells versus supernatant, and replace-media experiments, growth kinetics were performed by inoculating HIEs with human norovirus at $3.3–9.3 \times 10^5$ copies/well. Differentiated HIE monolayers (J3 line) grown in triplicate wells of a 96-well plate were inoculated with GII.1, GII.2, GII.3, or GII.4 Sydney strains. After a 1-hour incubation at 37°C, 5% CO_2, plates were washed twice with CMGF$^-$ and 100 μL of differentiation medium was added. For each set of infections, 1 plate was immediately frozen at −70°C and the remaining plates were incubated at 37°C, 5% CO_2 for the length of the experiment. Plates were frozen at 12, 24, 48, and 72 hpi. An extra plate at 96 hpi was included for the replace-media experiment. For growth curves, we extracted RNA from frozen lysates (cells and supernatant). For cells versus supernatant experiments, we removed supernatants before cells were harvested at each time point. For the replace-media experiment, we collected cell culture supernatants and replaced them with an equal volume of fresh differentiation media at each time point. Viral RNA levels in frozen lysates, cells, or supernatants were determined by RT-qPCR. The input virus used for each experiment is described in each figure legend.

Infectious Dose 50% (ID_{50})

Determination of ID_{50} was performed by inoculating HIEs with 10-fold serial dilutions of human norovirus. Differentiated HIEs monolayers (J3 line) grown in 6 wells of a 96-well plate were inoculated with 10-fold serial dilutions of GII.3, GII.4 Den Haag, or GII.4 Sydney. After a 1-hour incubation at 37°C, 5% CO_2, plates were washed twice with CMGF$^-$ and 100 μL of differentiation medium was added. For each set of infections, 1 plate was immediately frozen at

–70°C and the remained plates were incubated at 37°C, 5% CO_2 for 72 hours. We determined viral RNA levels in frozen lysates, cells, or supernatants by RT-qPCR. Wells that showed virus replication as an increase in genomic copies per well at 72 hours versus 1 hpi were scored as positive. ID_{50} was calculated by the Reed-Muench method (*9*).

Inactivation Treatments

Alcohol Treatment

We diluted 10% fecal filtrates 1:10 in 70% ethanol or isopropanol solutions. After incubation for 1 minute or 5 minutes, we neutralized the samples by 1:10 dilution in $CMGF^{-}$ supplemented with 10% FBS. A nontreatment control (10% fecal filtrate diluted 1:100 in PBS) and a neutralization control (10% fecal filtrate diluted 1:10 in PBS and 1:10 in $CMGF^{-}$ supplemented with 10% FBS) were also included. All alcohol solutions were made fresh by addition of the appropriate volume of cell culture grade water (Life Technologies) to molecular biology grade absolute ethanol or isopropanol (Fisher Scientific, Fairlawn, NJ, USA).

Chlorine Suspension Assays

We prepared fresh chlorine stock solutions at 1,000 ppm and 10,000 ppm by dilution of commercial bleach (Clorox, 6% sodium hypochlorite) in cell culture grade water (Life Technologies). For chlorine inactivation experiments, we diluted 20 μL of 10% fecal filtrate in an appropriate volume of 10,000 ppm or 1,000 ppm stock solutions to achieve final chlorine concentrations of 5000, 1000, 800, 600, 400, 200, 100, 50, and 5 ppm. After exposure for 1 minute, we added sodium thiosulfate (final concentration: 50 mg/L) to neutralize free chlorine. A nontreatment control (10% fecal filtrate diluted in PBS) and a neutralization control (10% fecal filtrate diluted in PBS and sodium thiosulfate) were also included. All incubations were performed at room temperature. All inactivation experiments and infections were done in triplicate.

Statistical Analysis

All statistical analyses were performed using GraphPad Prism 7.0 (GraphPad Software, La Jolla, CA, USA). Experiments were performed at least 3 times (3 technical replicates each time) from independent enteroid preparations, as indicated in the figure legends. Data are presented as mean ± SD. Except when specified, a Student's *t* test was used to determine statistical significance. Specific p values are detailed in the figure legends.

References

1. Saxena K, Blutt SE, Ettayebi K, Zeng XL, Broughman JR, Crawford SE, et al. Human intestinal enteroids: a new model to study human rotavirus infection, host restriction, and pathophysiology. J Virol. 2016;90:43–56. http://dx.doi.org/10.1128/JVI.01930-15

2. Zou WY, Blutt SE, Crawford SE, Ettayebi K, Zeng XL, Saxena K, et al. Human intestinal enteroids: new models to study gastrointestinal virus infections. Methods Mol Biol. 2017. http://dx.doi.org/10.1007/7651_2017_1

3. Schmittgen TD, Livak KJ. Analyzing real-time PCR data by the comparative C(T) method. Nat Protoc. 2008;3:1101–8. http://dx.doi.org/10.1038/nprot.2008.73

<eref>4. U.S. Department of Health and Human Services, Office of Science and Health Reports, DRR/NIH. Guide for the care and use of laboratory animals. DHHS pub. no. (NIH) 85–23, revised 1985. https://olaw.nih.gov/resources/publications.</eref>

5. Cannon JL, Barclay L, Collins NR, Wikswo ME, Castro CJ, Magaña LC, et al. Genetic and epidemiologic trends of norovirus outbreaks in the United States from 2013 to 2016 demonstrated emergence of novel GII.4 recombinant viruses. J Clin Microbiol. 2017;55:2208–21. http://dx.doi.org/10.1128/JCM.00455-17

6. Vega E, Barclay L, Gregoricus N, Shirley SH, Lee D, Vinjé J. Genotypic and epidemiologic trends of norovirus outbreaks in the United States, 2009 to 2013. J Clin Microbiol. 2014;52:147–55. http://dx.doi.org/10.1128/JCM.02680-13

7. Kroneman A, Vennema H, Deforche K, v d Avoort H, Peñaranda S, Oberste MS, et al. An automated genotyping tool for enteroviruses and noroviruses. J Clin Virol. 2011;51:121–5. http://dx.doi.org/10.1016/j.jcv.2011.03.006

8. Vinjé J. Advances in laboratory methods for detection and typing of norovirus. J Clin Microbiol. 2015;53:373–81. http://dx.doi.org/10.1128/JCM.01535-14

9. Reed LJ, Muench H. A simple method of estimating fifty percent endpoints. Am J Hyg. 1938;27:493–97. http://dx.doi.org/10.1093/oxfordjournals.aje.a118408

Technical Appendix Table. RT-qPCR primers and probes to analyze gene expression levels for markers of differentiated and undifferentiated small intestinal epithelial cell types

Cell type	Target	Gene symbol	Assay reference	Source
Stem cells	Leucin-rich-repeat-containing G-protein-couple receptor 5	LGR5	Hs00969422_m1	Life Technologies
	Marker of proliferation Ki-67	MKI67	Hs.PT.58.38933492	Integrated DNA Technologies
Enterocytes	Sucrose isomaltase	SI	Hs00356112_m1	Life Technologies
	Intestinal-type alkaline phosphatase	ALPI	Hs00357579_g1	Life Technologies
Enteroendocrine cells	Chromogranin A	CHGA	Hs00900370_m1	Life Technologies
	Synaptophysin	SYP	Hs.PT.58.27207712	Integrated DNA Technologies
	Free fatty acid receptor 4	FFA4R	Hs00699184_m1	Life Technologies
Goblet cells	Mucin 2	MUC2	Hs.PT.58.4321237	Integrated DNA Technologies
	Trefoil factor 3	TFF3	Hs00902278_m1	Life Technologies
Paneth cells	Defensin α 5	DEFA5	Hs.PT.58.39503641	Integrated DNA Technologies
	Lyzosime	LYZ	Hs.PT.58.24761205	Integrated DNA Technologies
All cells	Glyceraldehyde-3-phosphate dehydrogenase	GADPH	Hs02758991_g1	Life Technologies

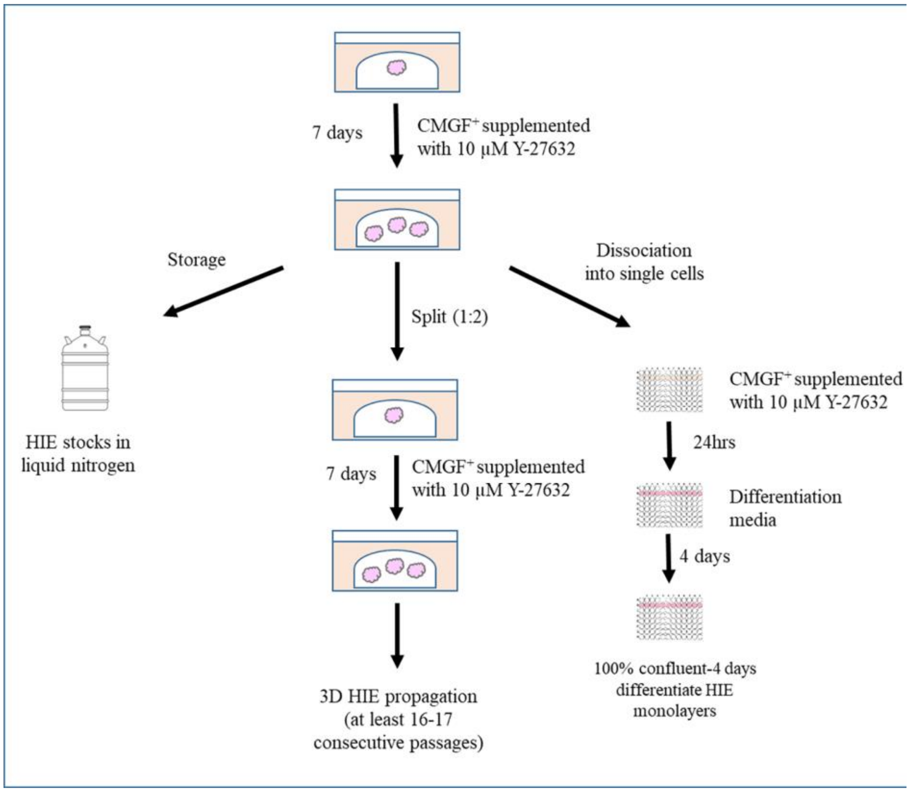

Technical Appendix Figure 1. Human intestinal enteroid culture (propagation and differentiation). Jejunal HIE cultures (J2 or J3 lines) were grown as undifferentiated 3-dimensional (3D) cultures. HIEs were recovered from liquid nitrogen, suspended in 20 µL of Matrigel (≈40 crypts), plated in a single well of a 24-well plate, and grown as 3D cultures in CMGF$^+$ medium supplemented with 10 µM Y-27632 (Sigma). The medium was replaced every 48 hours. After 7 days, highly dense 3D cultures were either split 1:2 and embedded in Matrigel, frozen in liquid nitrogen for further use, or dissociated into single cell suspensions and plated as undifferentiated monolayers, as described previously (*2*).

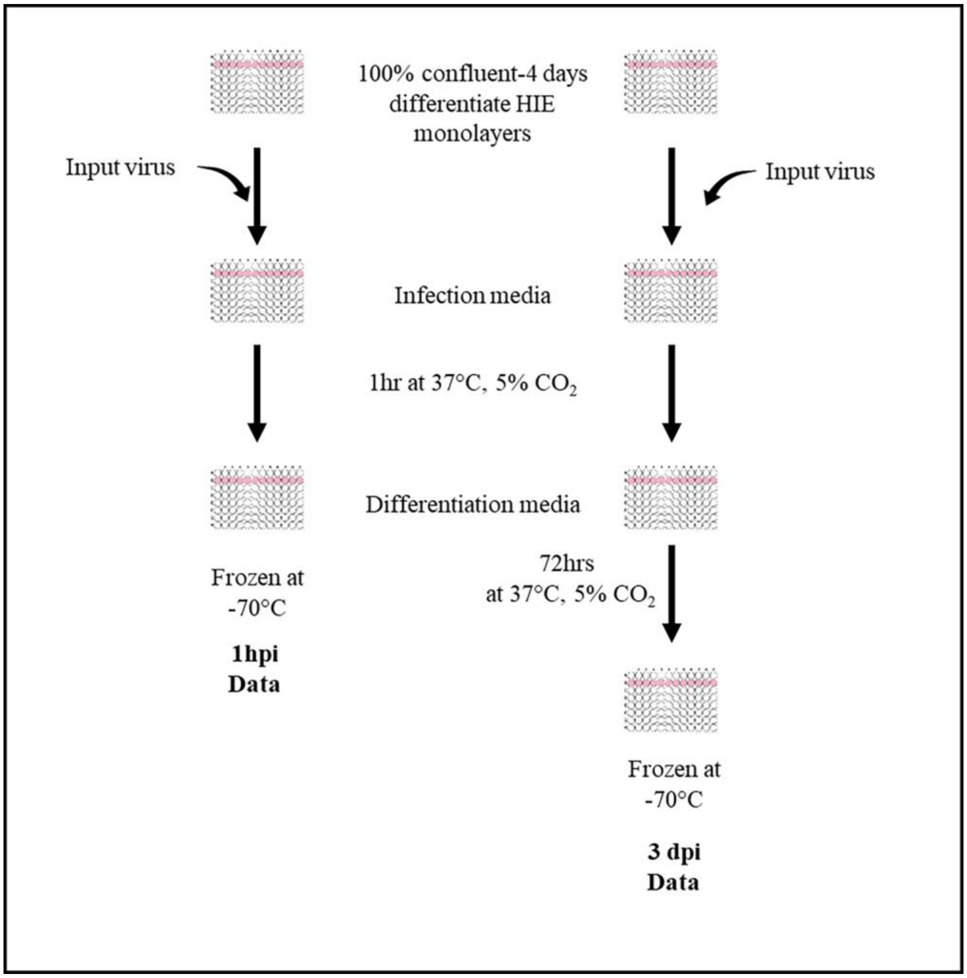

Technical Appendix Figure 2. To determine viral infectivity, duplicate plates were inoculated. After 1 hour of incubation at 37°C and 5% CO_2, monolayers were washed twice with CMGF$^-$ and 100 μL of differentiation medium containing 1% sow bile, 500 μM GCDCA, or 500 μM GCDCA plus 50 μM ceramide was added to each well. For each set of infections, 1 plate was immediately frozen at –70°C and a duplicate plate was incubated at 37°C, 5% CO_2 for 72 hours and frozen at –70°C. Viral RNA was extracted from input virus and HIE monolayers at 1 hour postinfection and 3 days postinfection were determined by RT-qPCR.
